# So What?

**DOI:** 10.31486/toj.24.5046

**Published:** 2024

**Authors:** Bobby D. Nossaman

**Affiliations:** Department of Anesthesiology and Perioperative Medicine, Ochsner Clinic Foundation, New Orleans, LA and The University of Queensland Medical School, Ochsner Clinical School, New Orleans, LA

*The graduate student made an eloquent research presentation that included detailed statistical analyses. The wizened professor asked one question: “So what?”*

## INTRODUCTION

Historically, the statistical approaches we use to assess the results from clinical studies have been based upon the concept of null hypothesis significance testing.^1^ The frequentist tests—*t* tests, chi-square tests, analysis of variance, linear and logistic regression analyses—are used with the assumption that the null hypothesis is true (no difference observed between the parameters of interest). This assumption then allows selection of the alternate hypothesis once the calculation of the frequentist test statistic falls below a preassigned cut point, usually *P* <0.05.^2^ However, these tests can be misleading in suggesting a clinical effect, as frequentist tests do not provide 2 important pieces of information: the magnitude of the effect of the intervention and the precision of that effect.^1,3^

Clinicians want to apply the best information obtained from clinical studies. However, when medical researchers only use frequentist tests to investigate their results, statistically significant results may or may not have clinical importance.^3^ Rather than using frequentist analyses, researchers should examine the degree of clinical difference with measures of effect size—risk or proportion differences in bivariate analyses and adjusted or standardized risk differences in multivariable analyses—and then determine if those differences are clinically important.^3-5^ We demonstrate the test differences on the same dataset used in Chai et al^6^ with the use of additional data collected but not reported in the main article.

## EXAMPLE

Concerns have long been expressed that epidural analgesia may delay the process of maternal labor, leading to an increase in the incidence of instrumental delivery including the need for cesarean.^7-9^ Over the years, a low-dose epidural analgesia technique^10-13^ has been developed and was used for patients included in the study by Chai et al.^6^ We examined the association of the duration of epidural labor analgesia (in hours) to the incidence of instrumental delivery, first using frequentist testing and then using risk differences to analyze the same data.^4,5^

Chi-square analysis with the classical statistical significance cut point of *P* <0.05^2^ showed that the duration of epidural labor analgesia was statistically associated with the incidence of instrumental delivery (chi-square=6.5, *P*=0.0110). The blue incidence line in the Figure increases

during the time period of interest and leads us to suspect a clinical association exists because the *P* value is less than the traditional cut point of 0.05.^2^

**Figure.**
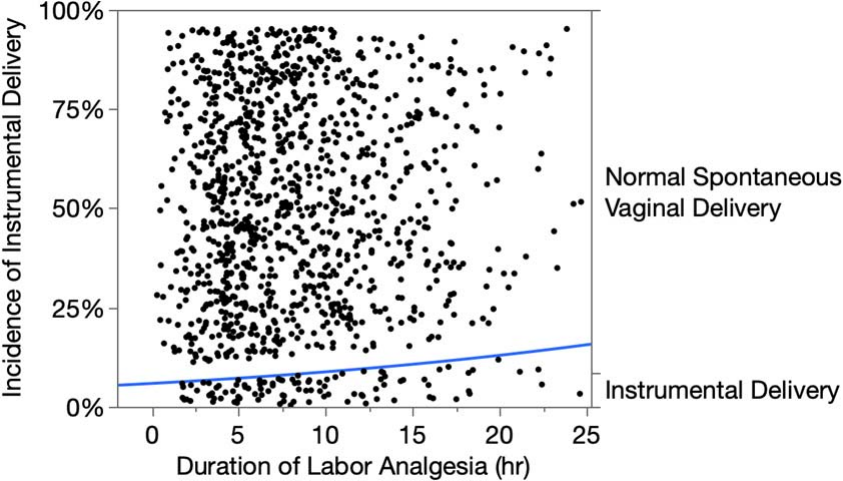
**Logistic fit plot of the incidence of instrumental delivery (blue line) associated with the duration of epidural labor analgesia in 1,231 parturients undergoing a trial of labor. Chi-square=6.5, *P*=0.0110.** hr, hour.

Using the same data set, we calculated the risk difference of the magnitude and precision of the duration of epidural labor analgesia with the incidence of instrumental delivery and obtained a mean risk value of 0.3% (CI 0.04%-0.6%) increase per hour of labor. As an example, the mean duration of labor was 8.5 hours, and the incidence of instrumental delivery increased from a baseline of 6.5% after the initial 2 hours of labor to an incidence of instrumental delivery of 8.7% after 10.5 hours of labor. This 2.2% change allows clinicians to interpret the importance of this association.

Additionally, we can investigate the interactions of additional clinical variables of interest by readjusting or standardizing their risk differences.^14^ We added delivery body mass index (BMI) to the analysis as it was the chief independent predictor of interest in Chai et al.^6^ The readjusted risk differences are shown in the Table. The addition of BMI to the model now increased the incidence of instrumental delivery to 0.4% (CI 0.1%-0.7%) per hour of labor, a minimal additive effect. The determination of whether this calculated effect size is clinically relevant depends upon the experience and professional practice of the clinician, as it should be.^4,5^

**Table.** Adjusted Risk Differences for Body Mass Index and Duration of Labor Analgesia and the Risk of Instrumental Delivery in 1,231 Parturients

Term	Standardized Risk Difference	95% CL	Std Error	Log-Ratio Chi-Square	*P* Value
Intercept	0.09	0.01 to 0.17	0.04	5.4	0.0207
Delivery body mass index	–0.001	–0.004 to 0.001	0.002	1.0	0.3261
Duration of labor analgesia	0.004	0.001 to 0.007	0.002	5.2	0.0221

Notes: *P* values <0.005 are statistically significant.^2^ The 95% CLs are Wald-based as the dataset is >1,000 rows.

CL, confidence limit; Std Error, standard error of the risk difference.

## CONCLUSION

Although *P* values obtained from frequentist tests may suggest a clinical effect, the value does not reveal the magnitude or the precision of that effect. The use of measures of effect size can quantify this clinical influence. Properly conducted research studies will improve our delivery of health care when they answer the clinically important question: “*So what?*”

## References

[1] Nakagawa S, Cuthill IC. Effect size, confidence interval and statistical significance: a practical guide for biologists [published correction appears in *Biol Rev Camb Philos Soc*. 2009 Aug;84(3):515]. Biol Rev Camb Philos Soc. 2007;82(4):591-605. doi: 10.1111/j.1469-185X.2007.00027.x 17944619

[2] Benjamin DJ, Berger JO, Johannesson M, Redefine statistical significance. Nat Hum Behav. 2018;2(1):6-10. doi: 10.1038/s41562-017-0189-z 30980045

[3] Schober P, Bossers SM, Schwarte LA. Statistical significance versus clinical importance of observed effect sizes: what do *P* values and confidence intervals really represent? Anesth Analg. 2018;126(3):1068-1072. doi: 10.1213/ANE.0000000000002798 29337724 PMC5811238

[4] Kim HY. Statistical notes for clinical researchers: effect size. Restor Dent Endod. 2015;40(4):328-331. doi: 10.5395/rde.2015.40.4.328 26587420 PMC4650530

[5] Kim HY. Statistical notes for clinical researchers: risk difference, risk ratio, and odds ratio. Restor Dent Endod. 2017;42(1):72-76. doi: 10.5395/rde.2017.42.1.72 28194368 PMC5300861

[6] Chai M, Vining A, Koveleskie J, Sumrall W, Nossaman BD. Risk of instrumental delivery in maternal obesity: estimates with measures of effect size. Ochsner J. 2024;24(3). doi: 10.31486/toj.24.0041 PMC11398620 39280873

[7] Kaminski HM, Stafl A, Aiman J. The effect of epidural analgesia on the frequency of instrumental obstetric delivery. Obstet Gynecol. 1987;69(5):770-773. 3574805

[8] Thorp JA, Hu DH, Albin RM, The effect of intrapartum epidural analgesia on nulliparous labor: a randomized, controlled, prospective trial. Am J Obstet Gynecol. 1993;169(4):851-858. doi: 10.1016/0002-9378(93)90015-b 8238138

[9] Walton P, Reynolds F. Epidural analgesia and instrumental delivery. Anaesthesia. 1984;39(3):218-223. doi: 10.1111/j.1365-2044.1984.tb07230.x 6703287

[10] Jouppila R, Jouppila P, Karinen JM, Hollmén A. Segmental epidural analgesia in labour: related to the progress of labour, fetal malposition and instrumental delivery. Acta Obstet Gynecol Scand. 1979;58(2):135-139. doi: 10.3109/00016347909154571 452866

[11] Sharma SK, Sidawi JE, Ramin SM, Lucas MJ, Leveno KJ, Cunningham FG. Cesarean delivery: a randomized trial of epidural versus patient-controlled meperidine analgesia during labor. Anesthesiology. 1997;87(3):487-494. doi: 10.1097/00000542-199709000-00006 9316951

[12] Bakhamees H, Hegazy E. Does epidural increase the incidence of cesarean delivery or instrumental labor in Saudi populations? Middle East J Anaesthesiol. 2007;19(3):693-704. 18044297

[13] Halpern SH, Muir H, Breen TW, A multicenter randomized controlled trial comparing patient-controlled epidural with intravenous analgesia for pain relief in labor. Anesth Analg. 2004;99(5):1532-1538. doi: 10.1213/01.ANE.0000136850.08972.07 15502060

[14] Naimi AI, Whitcomb BW. Estimating risk ratios and risk differences using regression. Am J Epidemiol. 2020;189(6):508-510. doi: 10.1093/aje/kwaa044 32219364

